# Evaluating the impact of a mandatory pre-abortion ultrasound viewing law: A mixed methods study

**DOI:** 10.1371/journal.pone.0178871

**Published:** 2017-07-26

**Authors:** Ushma D. Upadhyay, Katrina Kimport, Elise K. O. Belusa, Nicole E. Johns, Douglas W. Laube, Sarah C. M. Roberts

**Affiliations:** 1 Advancing New Standards in Reproductive Health (ANSIRH), Bixby Center for Global Reproductive Health, Department of Obstetrics, Gynecology and Reproductive Sciences, University of California, San Francisco, Oakland, California, United States of America; 2 Obstetrics and Gynecology, University of Wisconsin School of Medicine and Public Health, University of Wisconsin, Madison, Wisconsin, United States of America; National Academy of Medical Sciences, NEPAL

## Abstract

**Background:**

Since mid-2013, Wisconsin abortion providers have been legally required to display and describe pre-abortion ultrasound images. We aimed to understand the impact of this law.

**Methods:**

We used a mixed-methods study design at an abortion facility in Wisconsin. We abstracted data from medical charts one year before the law to one year after and used multivariable models, mediation/moderation analysis, and interrupted time series to assess the impact of the law, viewing, and decision certainty on likelihood of continuing the pregnancy. We conducted in-depth interviews with women in the post-law period about their ultrasound experience and analyzed them using elaborative and modified grounded theory.

**Results:**

A total of 5342 charts were abstracted; 8.7% continued their pregnancies pre-law and 11.2% post-law (p = 0.002). A multivariable model confirmed the law was associated with higher odds of continuing pregnancy (aOR = 1.23, 95% CI: 1.01–1.50). Decision certainty (aOR = 6.39, 95% CI: 4.72–8.64) and having to pay fully out of pocket (aOR = 4.98, 95% CI: 3.86–6.41) were most strongly associated with continuing pregnancy. Ultrasound viewing fully mediated the relationship between the law and continuing pregnancy. Interrupted time series analyses found no significant effect of the law but may have been underpowered to detect such a small effect.

Nineteen of twenty-three women interviewed viewed their ultrasound image. Most reported no impact on their abortion decision; five reported a temporary emotional impact or increased certainty about choosing abortion. Two women reported that viewing helped them decide to continue the pregnancy; both also described preexisting decision uncertainty.

**Conclusions:**

This law caused an increase in viewing rates and a statistically significant but small increase in continuing pregnancy rates. However, the majority of women were certain of their abortion decision and the law did not change their decision. Other factors were more significant in women’s decision-making, suggesting evaluations of restrictive laws should take account of the broader social environment.

## Introduction

In July 2013, a Wisconsin law took effect mandating that abortion providers display and describe the ultrasound image to patients prior to offering an abortion [1, 2]. While ten states have laws requiring abortion providers to offer women the opportunity to view their ultrasound images [3], Wisconsin’s law goes a step further by requiring them to present the image in the patient’s line of sight, whether or not the woman wishes to see it. If a woman does not want to view, she may physically turn her head away or close her eyes. The technician is required to give the woman a verbal description of the ultrasound image, including identification of fetal parts, the heartbeat and current development of the fetus. The law further requires abortion providers to offer patients a printed version of their ultrasound image and a state-produced booklet describing fetal development throughout pregnancy. Additionally, a woman must receive in-person state-directed information before starting a 24 hour waiting period and subsequently making a second trip to the clinic before she can have an abortion [1].

In recent decades, ultrasound use in abortion care has become routine, generally used to determine gestation of the pregnancy, multiple pregnancies, and other clinical indications [4–7]. Current medical guidelines note that while ultrasound is not required, it is recommended in abortion care [8]. Patient viewing of the pre-abortion ultrasound image, however, is not covered by medical guidelines. Viewing is an ancillary activity, not integral to the medical provision of abortion care. Laws mandating ultrasound viewing are not based on an identified medical need.

Drawing on experiences of patient ultrasound viewing in wanted pregnancies, scholars have speculated that ultrasound viewing prior to abortion would dissuade women from abortion [9–11]. However, recent literature on maternal-fetal bonding through ultrasound challenges the claim that viewing is central to bonding [12] and has posited ultrasound viewing as a social process rather than simple medical information [13, 14].

Research on the effects of offering voluntary ultrasound viewing on women’s experience with abortion [15–18] provides evidence that ultrasound viewing does not dissuade women from abortion. A recent analysis of over 15,000 visits to outpatient abortion care facilities where women were offered the option to view their pre-abortion ultrasound image found that 43% of the women chose to view the image [15, 17] and that, for the majority of women who viewed their ultrasound image, viewing did not affect their likelihood of proceeding to abortion; among women who viewed their ultrasound, 98.4% proceeded to abortion [15]. For the very small subset of women who reported low decision certainty, viewing did slightly increase the odds of continuing a pregnancy [15]. However, it was not possible to distinguish whether it was viewing the image that swayed the more uncertain women, or whether they chose to view the image in order to be swayed. Additional research shows that, aside from impact on decision-making, pre-abortion ultrasound viewing can have other effects on women [17–19]. In a study of 20 women interviewed after receiving an ultrasound as part of abortion care, women’s accounts illustrated that ultrasound viewing can cause emotional difficulty for women who plan to terminate their pregnancy [16], but other studies have found that the most common emotional response to ultrasound viewing is a neutral one, with many women reporting that viewing had no impact on them [19]. However, given findings that many women appreciate having the choice whether to view their ultrasound image [17, 20–22], it is unclear if we can generalize from findings on the effects (or lack thereof) of voluntary viewing to settings where viewing is mandatory, such as Wisconsin.

To date, three other state legislatures, Louisiana, Texas, and Kentucky have enacted similar laws mandating that abortion providers display and describe the ultrasound image prior to abortion. Two additional states, North Carolina and Oklahoma have passed similar laws, but they are enjoined by court order [3]. Other states may be considering similar mandatory viewing laws, making it important to examine the effects of such laws on women and their decisions to continue their pregnancies versus proceed to abortion.

We designed a mixed-methods study, integrating qualitative and quantitative methods, to investigate a mandatory ultrasound viewing law. We analyzed quantitative data abstracted from medical records from a high-volume abortion care facility in Wisconsin to examine whether this law impacted women’s decision to view their ultrasound image and/or their decision to proceed to abortion or continue the pregnancy. In parallel, we conducted in-depth interviews with women who sought abortion care at the same facility and were subject to the law to qualitatively explore their decision-making about viewing and, for those who viewed, perceived effects of viewing.

## Methods

We abstracted medical chart data and recruited participants for in-depth interviews at a high-volume abortion-providing facility in Milwaukee, Wisconsin. Over the course of data collection and analysis, the study team met frequently to discuss findings iteratively to inform ongoing analyses of both the qualitative and the quantitative data. The University of California, San Francisco Committee on Human Research granted ethical approval for all research protocols (Medical Chart Data: original approval date: 10 March 2015; study number: 15–15830; Interview Data: original approval date: 24 March 2015; study number: 15–15770).

Both before and after the law was implemented, all patients at the facility received a pre-abortion ultrasound on their first visit, along with blood testing, counseling, and other intake procedures. The main change after the law’s implementation was related to the display of the ultrasound image: prior to the law’s implementation, viewing the ultrasound image was voluntary; after the law went into effect, the ultrasound screen was placed in patients’ direct line of sight. Wisconsin state law already required a 24-hour waiting period between a patient’s initial information visit and the actual procedure. Therefore, both before and after implementation of the ultrasound viewing law, patients had to wait at least 24 hours after they received the ultrasound before they could have the abortion.

### Medical chart data

Quantitative data came from patient medical charts, abstracted by two facility staff and one UCSF research assistant. Staff abstracted sociodemographic and clinical chart data for all abortion patients from one year prior to the law’s implementation (July 7, 2012 –July 6, 2013) to one year after the law’s implementation (July 7, 2013 –July 6, 2014). Typically, only one ultrasound is performed per abortion, however some women had additional ultrasound visits, which were also abstracted.

#### Outcome variables

The main outcome of interest was continuing a pregnancy, defined as being eligible for an abortion but not obtaining one at the study clinic. Women who were eligible for an abortion but did not return were considered to have continued their pregnancy because it was not possible to assess whether they had an abortion at a nearby facility or elsewhere.

#### Main Independent variable

For all analyses, the primary independent variable was time period pre- or post-law.

#### Potential mediator

**Fig 1:**
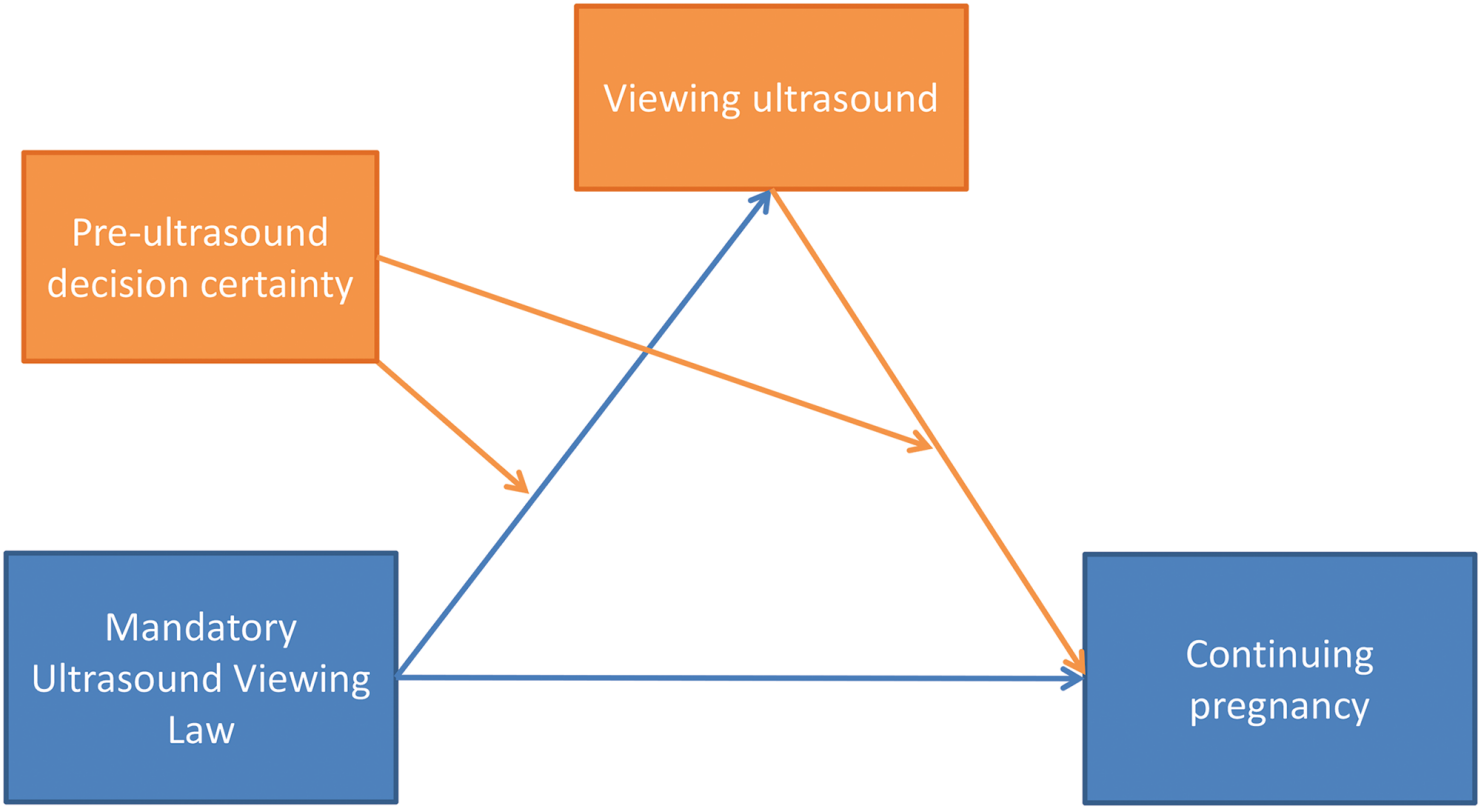
Conceptual model.

Per our conceptual model (Fig 1), we wanted to assess whether changes in viewing the ultrasound image either on the screen or receiving a printout of the image mediated the effects of the law on the decision to continue the pregnancy. In the pre-law period, all women seeking an abortion were offered the opportunity to view the image on the screen and in the post-law period women were able to look away if they did not want to see. In both the pre-law and post-law periods, all women were offered a printout of the ultrasound image. In both periods, whether the patient viewed the ultrasound image and whether she received a printout was recorded in her chart. For all analyses, we used a single dichotomous variable, combining both items for whether the woman viewed the image on screen or received the printout. As has been done in previous studies [15, 17], when women had more than one ultrasound, they were assigned to the ‘viewed ultrasound’ designation if they had viewed or received a printout of at least one ultrasound.

#### Potential moderator

We also examined decision certainty as a moderator of the association between the law and viewing the ultrasound image. The interviews in this study and previous quantitative research [17] have found decision certainty to be a key factor in whether the woman views her ultrasound image. Thus, we hypothesized that decision certainty would moderate the effect between the law and viewing. Previous research has also found that decision certainty is also associated with decisions to have an abortion [15, 23, 24]. Thus, we hypothesized that it would also moderate the effect of viewing the ultrasound on the main outcome of continuing the pregnancy (Fig 1). Counselors assessed each woman’s certainty about her decision to have an abortion at the clinic prior to the ultrasound and then documented whether she was firm or uncertain on an intake form. If the woman was uncertain, counselors would talk more with her and make notes on reasons for uncertainty. In some cases, women were referred out for further counseling and not scheduled for an abortion. Because there were so few women with low decision-certainty and with missing certainty data, we combined these groups in our analyses.

#### Control variables

We controlled for several other potential variables that previous studies [15, 17] suggested could be related to women’s viewing decisions or decisions to continue a pregnancy. These included sociodemographic characteristics (age, education, race/ethnicity, marital status, partner relationship, urbanicity), parity, time since last pregnancy, the primary decision-maker in the relationship (self, parent, husband, boyfriend, or other), support person presence at visit (yes, no), and noted concurrent or complicating medical issues.

Additionally, we included funding as a dichotomous variable, whether the woman qualified for partial financial assistance from an abortion fund or whether she was required to pay the full costs out of pocket. Women were interviewed about their ability to pay and qualification for funds at the ultrasound visit. After qualification was confirmed, women were referred to one or more abortion funds, which determined the amount the fund would provide. Some women received partial funding to cover laboratory costs from their ultrasound visit, even if they did not proceed to abortion. In post-hoc analyses, we assessed whether qualifying for such funding was significantly associated with both decision certainty and continuation of pregnancy. Thus we conducted additional analyses to better understand the effects of qualifying for funding on the outcome of interest.

We used the data directly as recorded in the chart, with the following few exceptions. We created an urban/rural designation based on zip code and Rural-Urban Commuting Area (RUCA) codes [25]. Entries in ‘other’ categories which fit a response group were recategorized (e.g. other for race/ethnicity). Data that were reported as continuous measures were grouped into categories for ease of analysis for age, highest level of education, and number of previous births. Dates which were written in error were recategorized as not in chart (e.g. if a woman entered her LMP as her date of last birth).

#### Analysis

We described socio-demographic and clinical characteristics of the samples before and after implementation of the law and assessed any differences from before to after the law using chi-square tests. We described and graphed the monthly rate of viewing the ultrasound image before and after the law. We also tested the hypothesized moderation of the effect of law on ultrasound viewing by decision certainty (Fig 1) by constructing an individual level model with an interaction between law and decision certainty.

We then described the proportion of women in the sample who did not have an abortion at the facility and the known reasons. Women who were not pregnant at the ultrasound visit, with no gestational sac seen, or who miscarried or probably miscarried were excluded from the subsequent analyses. Additionally, we excluded from subsequent analyses those who could not have an abortion at the facility because they were beyond the clinic’s gestational limit at the time of first ultrasound, beyond the height and weight limit, had an ectopic pregnancy, had a chronic medical condition, were on specific medications, were referred for services elsewhere, or were otherwise ineligible for care at the facility.

We then constructed a series of models, using a block modeling approach which incrementally added variables to examine the changes in the odds of continuing the pregnancy; we started with a simple model of law as the sole predictor and then added viewing and decision certainty.

Then we constructed adjusted multivariable models examining the effects of the law on the decision to continue pregnancy. The second multivariable model omitted viewing as a potential mediator. We also tested potential interactions between decision certainty and law and gestational age and law based on a priori hypotheses and post-hoc potential associations based on statistical significance. We conducted a post-hoc analysis of qualifying for abortion funds and decision certainty. For all models, we used generalized estimating equations with logistic regression specifications and the patient as the panel variable to account for multiple pregnancies among the same woman.

Based on the literature, we expected to find mediation and moderation among the law, viewing the ultrasound image, decision certainty, and continuation of pregnancy (Fig 1). To understand the effect of the law on viewing and viewing on continuation of pregnancy, we conducted a mediation analysis using Preacher & Hayes bootstrapped test of mediation [26].

We then sought to examine moderation. Our initial plan was to conduct a moderated mediation analyses. However, there were too few women who were uncertain who did not view in the post-law period for this or even stratified models to be appropriate. We therefore examined rates of continuing pregnancy stratified by decision certainty and viewing status.

Finally, we wanted to consider alternative plausible hypotheses to rule out other factors that may have been occurring around the time of the law change. Specifically, we sought to examine whether there were any underlying time trends that may be occurring unrelated to the law change, but that might explain our findings. Thus we conducted an interrupted time series analysis using segmented regression for continuing the pregnancy [27, 28]. We conducted a second segmented regression model that adjusted for aggregated covariates. We assessed for autocorrelation using the Durbin-Watson statistic and examined the data for evidence of seasonality. We did not find evidence of autocorrelation or seasonality.

### In-depth interviews

To explore women’s experiences of mandatory ultrasound viewing, the second author conducted semi-structured, in-depth phone interviews. Women were recruited following their ultrasound visit. Patients were eligible if they were over 18, English-speaking, and had received an ultrasound as part of abortion care at the study facility. Recruitment took place between May and September of 2015. Most women were interviewed about one week after their ultrasound visit.

To recruit participants, following the ultrasound, the staff technician gave patients a study flyer, which described the study’s objective to examine women’s experience of the Wisconsin ultrasound law and included a toll-free phone number for UCSF research staff. Interested potential participants called the number and were screened for eligibility. Eligible callers were verbally consented and, if interested, scheduled for a phone interview. As we neared saturation, we noticed a disproportionate representation of white respondents compared to the facility’s client population. We conducted a second round of recruitment purposively sampling women of color. After preliminary analyses, we found the experiences of women of color did not differ from white women. We thus determined that we had reached saturation soon after the second round of recruitment and ceased recruitment.

Phone interviews were conducted by the second author, a sociologist and expert in qualitative data collection and analysis. Respondents were asked about their experience at the facility on the day of their ultrasound, including ultrasound experience, what they thought and felt about viewing or not viewing the image and about hearing a description of the image, and any perceived impacts of viewing. Interviews were recorded and transcribed verbatim by a professional transcription company. In presenting the data below, we refer to respondents using pseudonyms.

Interviews averaged about 60 minutes. Respondents were compensated for their time with a $50 gift card.

#### Analysis of in-depth interviews

Interviews were analyzed by the second author in Atlas.ti 7 using elaborative coding, with general codes developed based on the research question for respondents’ emotional experience of viewing and their pregnancy decision-making and on findings from the quantitative data. Because the chart abstraction occurred simultaneously with the in-depth interviews, and thus the quantitative findings did not inform the interview guide itself, the ability of the in-depth interviews to shed light on some of the more unexpected quantitative findings was limited. Excerpts for these codes were detail-coded using modified grounded theory [29] to elucidate patterns within these broader experiences and decisions. Findings were discussed and contextualized by several authors, compelling an additional round of coding by the second author. Coding was considered complete when no new codes emerged.

## Results

### Medical chart data

Among all medical chart data requested from the 24 months of the study (N = 5595), 120 charts were missing (2.1%): 55 charts in the pre-law period and 65 charts in the post-law period. An additional 133 charts were outside referrals where the ultrasound was done at an alternate site and thus, the charts were not abstracted. Ultimately, 5342 charts were abstracted, with 2724 ultrasound visits in the pre-law period and 2618 in the post-law period.

There were slight differences between the pre- and post-law samples (Table 1). In the post-law period, women were less likely to report decision-making was shared with another person (3.7% pre-law vs 2.2% post-law, p = 0.001), and more likely to live in a rural zip code (6.1% pre-law vs 7.8% post-law, p = 0.02) than women in the pre-law period. Decision certainty was consistent between pre and post-law periods (Table 1).

**Table 1:** Characteristics of the pre- and post-law populations at a WI clinic, 7/7/2012-7/6/2014.

	Pre-Law	Post-Law	Total	Significant difference pre vs post?
N, #	2724	2618	5342	
Age, # (%)				N.S.
<20	289 (10.6)	293 (11.2)	582 (10.9)	
20–24	907 (33.3)	831 (31.7)	1738 (32.5)	
25–29	749 (27.5)	725 (27.7)	1474 (27.6)	
30–39	670 (24.6)	675 (25.8)	1345 (24.2)	
40+	109 (4.0)	93 (3.6)	202 (3.8)	
Not in chart	0 (0)	1 (<0.1)	1 (<0.1)	
Highest level of education, # (%)				N.S.
Less than High School	476 (17.5)	427 (16.3)	903 (16.9)	
High school diploma or GED	570 (20.9)	522 (19.9)	1092 (20.4)	
Associates degree / <4 yrs college	1067 (39.2)	1074 (41.0)	2141 (40.1)	
Bachelors degree or higher	547 (20.1)	543 (20.7)	1090 (20.4)	
Not in chart	64 (2.3)	52 (2.0)	116 (2.2)	
Race/Ethnicity, # (%)				N.S.
White	1192 (43.8)	1180 (45.1)	2372 (44.4)	
Black	1041 (38.2)	961 (36.7)	2002 (37.5)	
Latina	239 (8.8)	239 (9.1)	478 (8.9)	
Asian/Pacific Islander	107 (3.9)	104 (4.0)	211 (3.9)	
Other/mixed race	81 (3.0)	85 (3.2)	166 (3.1)	
Not in chart	64 (2.3)	49 (1.9)	113 (2.1)	
Funding, # (%)				p<0.001
Qualified for abortion funds^1^	1269 (46.6)	1052 (40.2)	2321 (43.4)	
Required to pay fully out of pocket^2^	1455 (53.4)	1566 (59.8)	3021 (56.6)	
Urbanicity based on zip code, # (%)				p = 0.02
Urban	2526 (92.7)	2371 (90.6)	4897 (91.7)	
Rural	166 (6.1)	201 (7.8)	369 (6.9)	
Not in chart	32 (1.2)	44 (1.7)	76 (1.4)	
Marital status, # (%)				p = 0.01
Never married	2097 (77.0)	2057 (78.6)	4154 (77.8)	
Married	318 (11.7)	281 (10.7)	599 (11.2)	
Divorced/Separated/Widowed	278 (10.2)	270 (10.3)	548 (10.3)	
Not in chart	31 (1.1)	10 (0.4)	41 (0.8)	
Partner’s relationship, # (%)				N.S.
Husband	281 (10.3)	239 (9.1)	520 (9.7)	
Boyfriend/Fiancé	1425 (52.3)	1411 (53.9)	2836 (53.1)	
Friend	522 (19.2)	511 (19.5)	1033 (19.3)	
Ex-husband/Ex-boyfriend	77 (2.8)	68 (2.6)	145 (2.7)	
Other/Not in chart	419 (15.4)	389 (14.9)	808 (15.1)	
Dominant decision-maker, # (%)				p = 0.001
Self	2483 (91.2)	2445 (93.4)	4928 (92.3)	
Parent/Guardian	30 (1.1)	32 (1.2)	62 (1.2)	
Husband/Fiancé/Boyfriend	10 (0.4)	18 (0.7)	28 (0.5)	
Shared between self and another	100 (3.7)	57 (2.2)	157 (2.9)	
Other/Not in chart	101 (3.7)	66 (2.5)	167 (3.1)	
Number of previous births, # (%)				N.S.
0	992 (36.4)	1003 (38.3)	1995 (37.3)	
1 or more	1485 (54.5)	1371 (52.4)	2856 (53.5)	
Not in chart	247 (9.1)	244 (9.3)	491 (9.2)	
Support person present, # (%)				N.S.
Yes	1012 (37.2)	966 (36.9)	1978 (37.0)	
No/Not in chart	1712 (62.8)	1652 (63.1)	3364 (63.0)	
Weeks gestation at first US visit, # (%)				N.S.
Less than 9 weeks	1828 (67.1)	1733 (66.2)	3561 (66.7)	
9–14 weeks	510 (18.7)	555 (21.2)	1065 (19.9)	
>14 weeks	378 (13.9)	326 (12.5)	704 (13.2)	
Not in chart^3^	8 (0.3)	4 (0.2)	12 (0.2)	
Multiple pregnancy, # (%)				N.S.
Yes	48 (1.8)	53 (2.0)	101 (1.9)	
No	2676 (98.2)	2565 (98.0)	5241 (98.1)	
Decision certainty at ultrasound visit, # (%)				N.S.
Firm	2548 (93.5)	2441 (93.2)	4989 (93.4)	
Uncertain	84 (3.1)	72 (2.8)	156 (2.9)	
Not in chart	92 (3.4)	105 (4.0)	197 (3.7)	
Viewed any ultrasound, # (%)				p<0.001
Viewed	1671 (61.3)	2381 (90.9)	4052 (75.9)	
Refused	1034 (38.0)	205 (7.8)	1239 (23.2)	
Not in chart	19 (0.7)	32 (1.2)	51 (1.0)	
Accepted a printout of the ultrasound image, # (%)				p = 0.01
Accepted	1050 (38.5)	971 (36.9)	2021 (37.8)	
Did not accept	1657 (60.8)	1608 (63.1)	3265 (61.1)	
Not in chart	17 (0.6)	39 (1.5)	56 (1.0)	
Continued the pregnancy, # (%)^4^				p = 0.002
Yes	228 (8.7)	284 (11.2)	512 (9.9)	
No	2397 (91.3)	2249 (88.8)	4646 (90.1)	

N.S. = Not statistically significant at p<0.05

^1^ Includes 2 women (1 pre-law and 1 post-law) who used Medicaid

^2^ Includes 2 women (1 pre-law and 1 post-law) who used private insurance.

^3^ Includes 10 women (6 pre-law and 4 post-law) who were not pregnant at first ultrasound

^4^ Excludes 181 women who were not eligible for an abortion at the study site (see Table 2) and 3 women who were excluded due to missing data on missing chart data.

While the law required providers to display the ultrasound image, women were legally permitted to physically turn their heads away or close their eyes. Women in the post-law period were more likely to view the ultrasound image than women in the pre-law period, although more than half of patients did choose to view pre-law (61.3% pre-law vs 90.9% post-law, p<0.001) (Table 1). Compared to the pre-law period, significantly fewer women qualified for assistance from an abortion fund in the post-law period (46.6% pre-law vs 40.2% post-law, p<0.001). Among those eligible for an abortion at the study site, women in the post-law period were more likely to continue with their pregnancy than women in the pre-law period (8.7% pre-law vs 11.2% post-law, p = 0.002).

#### Viewing the ultrasound image

**Fig 2:**
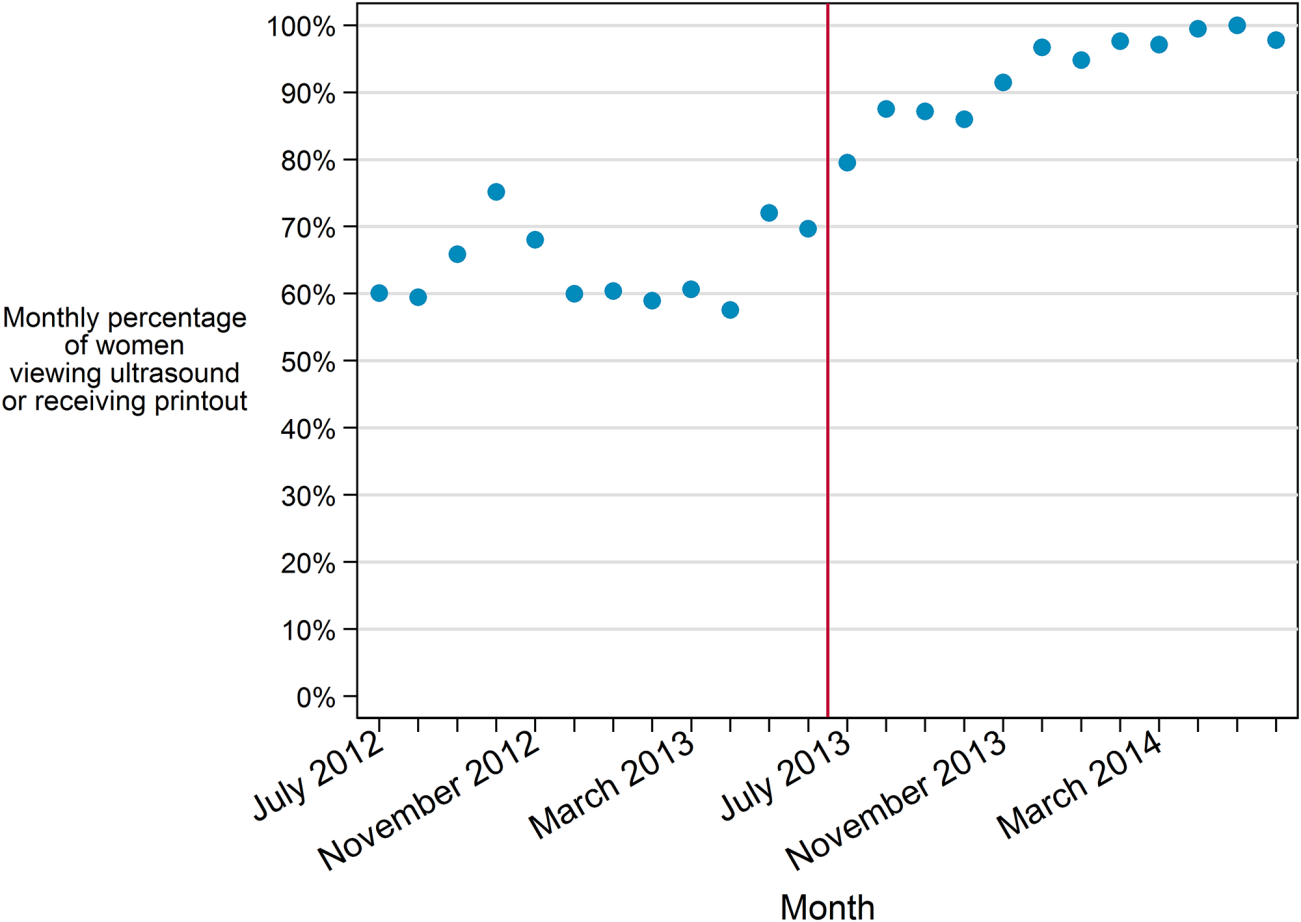
Monthly rate of viewing the ultrasound image or receiving printout.

Segmented regression analysis of the monthly aggregate data found a significant difference in viewing levels pre- vs post-law (p<0.001) (Fig 2). There was no significant time trend in percent of women viewing pre-law, but there was evidence of a statistically significant monthly increase in the post-law time period in the model (p = 0.02). In the individual-level model, we found no evidence of moderation of the effect of the law on viewing by decision certainty (interaction p = 0.10).

#### Continuing the pregnancy

Among the 5342 charts, 13% of women (695), did not proceed to abortion at the study site. Table 2 presents the women who did not have an abortion at the facility by reason. The most common reason women did not have an abortion at the facility was because they did not call or show for their abortion appointment or they called and cancelled or they never made an appointment in the first place (299, 43.0%) followed by reason not listed in chart (133, 19%).

**Table 2:** Primary reason for not having abortion (mutually exclusive) (total n = 693).

Total N	Pre-Law 325	Post-Law 368	Total 693	Included in continuing pregnancy analysis?
1. Not pregnant, no gestational sac seen, miscarriage, or possible miscarriage	37 (11.3%)	24 (6.5%)	61 (8.8%)	No
2. Ectopic, possible ectopic, or molar pregnancy (referred out)	12 (3.7%)	5 (1.4%)	17 (2.4%)	No
3. Chronic/high-risk medical condition (referred out)	2 (0.6%)	2 (0.5%)	4 (0.6%)	No
4. Current medications	0 (0%)	0 (0%)	0 (0%)	NA
5. Beyond height/weight limit	0 (0%)	0 (0%)	0 (0%)	NA
6. Anemic	0 (0%)	0 (0%)	0 (0%)	NA
7. Passed gestational limit at first ultrasound	46 (14.1%)	53 (14.4%)	99 (14.2%)	No
**Total**	**97**	**84**	**181**	
8. Referred elsewhere, other reason not listed above^1^	15 (4.6%)	7 (1.9%)	22 (3.2%)	Yes
9. Explicitly said she changed her mind^2^	23 (7.0%)	29 (7.9%)	52 (7.5%)	Yes
10. No call, no show for abortion appointment, called and cancelled without explanation, or did not schedule abortion appointment	134 (41.0%)	165 (44.8%)	299 (43.0%)	Yes
11. Other^3^	3 (0.9%)	3 (0.8%)	6 (0.9%)	Yes
12. Not listed in chart	53 (16.2%)	80 (21.7%)	133 (19.1%)	Yes
**Total**	**228**	**284**	**512**	

^1^ Includes patients who presented at 20–21 weeks and could not be seen before the gestational limit as well as patient desires for same day procedures, sedation, or referrals to clinics closer to their homes.

^2^ Includes 6 women who mentioned coercion or pressure to have an abortion.

^3^ Includes 1 or 2 women who did not have an abortion at the study site for the following reasons: cancelled due to financial reasons, wanting to go elsewhere for an abortion, not being able to continue the procedure due to pain.

For the main analyses, we excluded the 181 women (3.4% of all women) who were not eligible for an abortion at the facility, as well as one woman whose birthdate and age were missing, two women who left during the first appointment and were missing extensive substantial chart data and one missing data on weeks gestation from the continuing pregnancy analyses. We retained 512 women (9.6% of all women) in the dataset who did not have an abortion. Thus the subsequently analyzed dataset included 5158 charts, 2625 pre-law and 2533 post-law. This sample size afforded us statistical power of 90% to detect an increase of 3 percentage points or greater in the proportion of women who continued their pregnancy in the pre- and post-law periods, based on a baseline continuation rate of 12%.

Among the 5,158 women who were eligible for an abortion, 512 continued the pregnancy, 228 (8.7% of all eligible women) before the law and 284 (11.2% of all eligible women) after the law. A univariate model showing the effect of the law on continuing pregnancy is shown in Table 3 with additional variables added to the model. Introduction of the law was significantly associated with higher odds of continuing the pregnancy (Model A: OR = 1.33, 95% CI = 1.10–1.59). Recalling that the law required the image be displayed, but the woman could turn her head away or close her eyes, the effect of the law was no longer significant when viewing the ultrasound image was added to the model suggesting mediation; viewing was associated with a higher odds of continuing the pregnancy (Model B: AOR = 1.86, 95% CI = 1.41–2.45). The effect of viewing was attenuated after adding decision certainty to the model. Being uncertain about the abortion decision was associated with an 8-fold increase in the odds of continuing pregnancy, making it the strongest factor associated with continuing pregnancy (Model C: AOR = 8.11, 95% CI = 6.13–10.74).

**Table 3:** Time period, viewing ultrasound, decision certainty and their associations with continuing pregnancy, n = 5,158.

	A		B		C	
	OR	95% CI	AOR	95% CI	AOR	95% CI
Time period						
Pre-Law	Ref		Ref		Ref	
Post-Law	1.33**	1.10,1.59	1.13	0.93,1.38	1.17	0.96,1.43
Viewed or received printout of any ultrasound						
Refused			Ref		Ref	
Viewed			1.86***	1.41,2.45	1.66***	1.25,2.20
Not in chart			2.24	0.76,6.59	1.94	0.63,5.97
Decision certainty at ultrasound visit						
Firm					Ref	
Uncertain/Not in chart					8.11***	6.13,10.74

**p<0.01

***p<0.001

A full model, adjusted for covariates, demonstrates that the effects of viewing and decision certainty on continuing the pregnancy remain (Table 4). A final full model that omits viewing (a potential mediator) shows that the law was associated with a higher odds of continuing pregnancy (aOR = 1.23, 95% CI: 1.01–1.50). Being uncertain about the abortion decision (aOR = 6.39, 95% CI: 4.72–8.64) was also associated with continuing pregnancy. An interaction term between decision certainty and the law on the outcome of continuing pregnancy was not significant, suggesting that the law had a statistically significant effect among both firm and uncertain women.

**Table 4:** Factors associated with continuing pregnancy, with and without the mediator, viewing (n = 5,158).

	Model A—with the mediator		Model B—excluding the mediator	
	AOR	95% CI	AOR	95% CI
Time period				
Pre-Law	Ref	Ref	Ref	Ref
Post-Law	1.03	0.84,1.28	1.23*	1.01,1.50
Viewed or received printout of any ultrasound				
Refused	Ref	Ref	-	-
Viewed image	1.91***	1.42,2.55	-	-
Not in chart	1.83	0.58,5.79	-	-
Decision certainty at ultrasound visit				
Firm	Ref	Ref	Ref	Ref
Uncertain/Not in chart	5.97***	4.40,8.09	6.39***	4.72,8.64
Funding				
Qualified for abortion funds^1^	Ref	Ref	Ref	Ref
Required to pay fully out of pocket^2^	5.18***	4.01,6.69	4.98***	3.86,6.41
Age				
<20	1.06	0.74,1.52	1.09	0.76,1.56
20–24	Ref	Ref	Ref	Ref
25–29	0.93	0.72,1.21	0.92	0.71,1.20
30–39	0.85	0.64,1.13	0.83	0.62,1.10
40+	0.91	0.54,1.53	0.91	0.54,1.52
Highest level of education				
Less than high school	1.15	0.83,1.61	1.16	0.83,1.62
High school diploma or GED	Ref	Ref	Ref	Ref
Associates degree / <4 yrs college	1.00	0.76,1.31	1.00	0.76,1.31
Bachelors degree or higher	1.06	0.77,1.48	1.05	0.76,1.46
Not in chart	1.00	0.49,2.07	0.99	0.48,2.04
Race/Ethnicity				
White	Ref	Ref	Ref	Ref
Black	1.79***	1.40,2.29	1.89***	1.48,2.42
Latina	1.55*	1.09,2.20	1.58*	1.11,2.24
Asian/Pacific Islander	0.69	0.39,1.23	0.76	0.43,1.34
Other/mixed race	1.24	0.68,2.24	1.23	0.68,2.23
Not in chart	1.10	0.56,2.15	1.15	0.59,2.24
Urbanicity based on zip code				
Urban	Ref	Ref	Ref	Ref
Rural	0.86	0.57,1.31	0.86	0.57,1.30
Not in chart	1.11	0.50,2.49	1.05	0.47,2.35
Partner’s relationship				
Husband	Ref	Ref	Ref	Ref
Boyfriend/Fiancé	0.88	0.62,1.26	0.92	0.64,1.31
Friend	1.01	0.68,1.50	1.04	0.70,1.55
Ex-husband/Ex-boyfriend	0.54	0.26,1.15	0.55	0.26,1.18
Other/Not in chart	0.90	0.59,1.37	0.94	0.62,1.42
Dominant decision-maker				
Self	Ref	Ref	Ref	Ref
Parent/Guardian	0.91	0.39,2.14	0.88	0.38,2.05
Husband/Fiancé/Boyfriend	4.44**	1.77,11.15	4.56**	1.83,11.36
Shared between self and another	0.66	0.34,1.28	0.67	0.35,1.31
Other/Not in chart	1.52	0.92,2.49	1.51	0.92,2.48
Number of previous births				
0	Ref	Ref	Ref	Ref
1 or more	1.55***	1.21,1.98	1.53***	1.20,1.95
Not in chart	1.49*	1.05,2.11	1.51*	1.07,2.13
Support person present				
Yes	Ref	Ref	Ref	Ref
No/Not in chart	0.86	0.70,1.06	0.87	0.71,1.07
Multiple pregnancy				
Yes	1.34	0.72,2.48	1.39	0.75,2.58
No	Ref	Ref	Ref	Ref
Gestation at first US visit				
Less than 9 weeks	Ref	Ref	Ref	Ref
9–14 weeks	1.11	0.86,1.43	1.08	0.83,1.39
>14 weeks	2.24***	1.68,2.98	2.14***	1.61,2.84

^1^ Includes 2 women (1 pre-law and 1 post-law) who used Medicaid

^2^ Includes 2 women (1 pre-law and 1 post-law) who used private insurance

*p<0.05

**p<0.01

*** p<0.001

Additionally, women who were black and women who had at least one previous birth were more likely to continue their pregnancies than white women and nulliparous women. Women whose partners were the dominant decision-makers were also more likely to continue their pregnancies than those who identified themselves as the dominant decision-makers.

Women who were required to pay fully out of pocket were more likely to continue the pregnancy than those who qualified for partial assistance from an abortion fund (aOR = 4.98, 95% CI: 3.86–6.41). A further analysis of funding revealed that women who qualified for abortion funds were significantly more likely to be firm in their decision (98% of women who qualified for abortion funds were firm, compared to 94% of women who did not, p<0.001).

The results of the mediation analysis found that the relationship between the law and continuing the pregnancy was fully mediated by ultrasound viewing. The standardized regression coefficient between law and ultrasound viewing was statistically significant (0.30, p<0.001), as was the standardized regression coefficient between ultrasound viewing and continuing the pregnancy (0.09, p<0.001). We tested the significance of the indirect effect using bootstrapping procedures. The bootstrapped unstandardized indirect effect was 0.26, and the 95% confidence interval ranged from 0.019 to 0.033. Thus, the indirect effect was statistically significant. The direct effect was not significant (-0.005, p = 0.58), suggesting full mediation by viewing the ultrasound.

**Fig 3:**
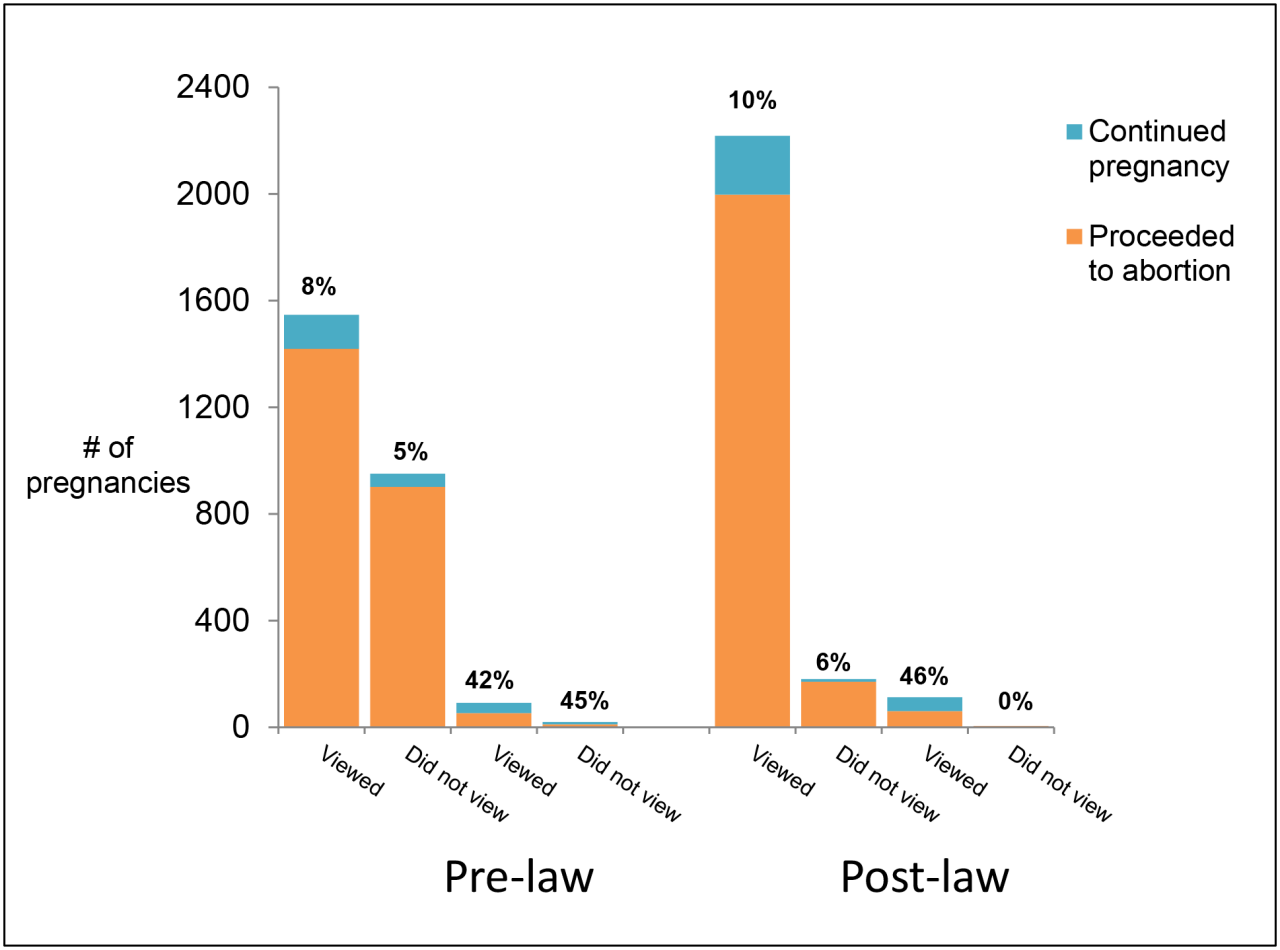
Number of pregnancies by time period, decision certainty, and viewing/picture receipt status and percent of women in each category continuing their pregnancy, n = 5127^A^. ^A^Excludes 181 women who were not eligible for abortion at the facility and 31 women for whom data on ultrasound viewing or picture receipt was missing.

After the ultrasound viewing law went into effect, the percentage of women continuing their pregnancies increased slightly in almost all groups stratified by decision certainty and viewing status (Fig 3). We wished to explore whether decision certainty moderated the relationship between the law and continuing pregnancy, but due to small numbers of women who were uncertain, we were unable to formally do so. We were, however, able to examine continuing pregnancy rates stratified by decision certainty (Fig 3). While the vast majority of women reported “firm” decision certainty and more than 90% of these women proceeded to abortion, there was a statistically significant increase in continuing pregnancy rates among women who were firm before and after the law (7.1% pre-law, 9.6% post-law, p = 0.002). Among the small proportion of women expressing uncertainty about their decision to have an abortion or who had missing data on decision certainty (4.5% of women eligible for abortion, n = 232), 43% of those pre-law (n = 49) and 44% of those post-law (n = 52) continued their pregnancy, a difference that was not statistically significant (p = 0.959).

**Fig 4:**
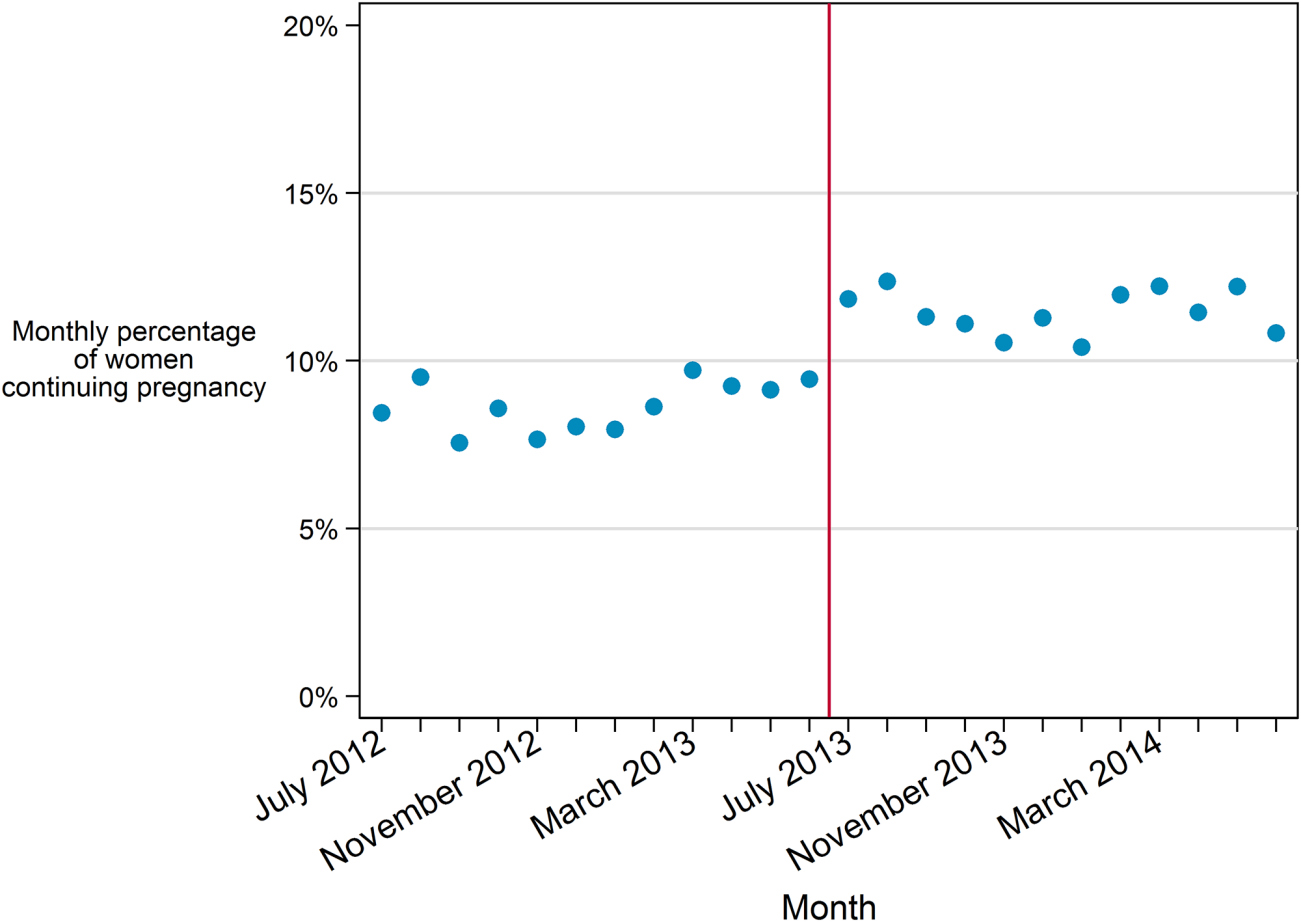
Adjusted monthly rate of continuing pregnancy, predicted value of segmented regression with covariates.

Results of the interrupted time series analysis found that overall, there was a slight but statistically significant increase in monthly continuing pregnancy rates over the entirety of the study time period (Table 5 Model A). Visually there appeared to be a significant increase at the time of the law and a significant difference in the monthly rate of women continuing pregnancies at the time the law was implemented supporting the individual-level results; however the difference did not reach statistical significance, perhaps due to lack of power [30]. Neither the slopes nor intercepts of pre- vs post-law linear fits of aggregate monthly data differed significantly (Table 5 Model B). We also tested the addition of monthly aggregate-level covariates for percent viewing and percent firm in decision; none were significant (Table 5 Model C, Fig 4).

**Table 5:** Monthly trend & segmented regression models of monthly continuing pregnancy rate.

	Coefficient	Standard error	P-value
A. Monthly trend only			
Month	0.002	0.001	0.022
Constant	0.077	0.011	0.000
B. Segmented regression model—simple			
Month	0.001	0.002	0.658
Law	0.023	0.021	0.293
Month since law	-0.001	0.003	0.732
Constant	0.080	0.016	0.000
C. Segmented regression model—with select covariates			
Month	0.001	0.002	0.662
Law	0.025	0.036	0.502
Month since law	-0.001	0.004	0.815
Viewing			
% of women refused to view or receive picture	Ref	Ref	Ref
% of women viewed or received picture	-0.010	0.129	0.939
% of women not in chart viewing	-0.442	1.001	0.665
Decision certainty			
% of women certain	Ref	Ref	Ref
% of women with uncertain or not in chart certainty	0.402	0.415	0.347
Funding			
% of women qualified for abortion funds	Ref	Ref	Ref
% of women required to pay fully out of pocket	-0.027	0.177	0.879
Constant	0.058	0.126	0.651

### In-depth interview findings

#### Characteristics of sample

A total of 23 women completed a semi-structured interview. Respondents ranged in age from 18 to 44 years old, with most (n = 13) in their 20s. Of the 23 women, 15 identified as white, 6 as black, 1 as multiracial (black and white), and 1 as Latina. Most (n = 16) had at least some college, six had high school degrees, and one left school after 11^th^ grade. At the time of their first appointment at the abortion clinic, most women were in the first trimester of their pregnancies: 14 were <7 weeks gestation, 6 were 7–13 weeks, and 3 were in the second trimester (13, 15, and 16 weeks, respectively). Broadly speaking, this sample was similar to the overall population that sought abortion care from the facility in the post-law period (Table 1). The exception to this similarity was in the relative over-participation of white women and under-participation of black and, particularly, Latina women.

The majority of respondents (n = 19) reported viewing their ultrasound image during their appointment at the abortion clinic. As the law mandates the visual display of the ultrasound image as well as a verbal description of the content of the image, the four women who declined to view did nonetheless hear a description of their ultrasound image. Most of the respondents (n = 18) were not aware of the law requiring that they be presented with their ultrasound image and listen to a description of the image. Three reported that they had a vague idea about the law prior to their appointment. One patient, for example, said she “might have read about it.” Just two respondents said that they knew about the law in advance of their first appointment, and both women explained that they learned of it in the course of doing research to prepare themselves for their abortion appointments; both women viewed their ultrasound image. Because of respondents’ general lack of knowledge about the law itself, and in light of the quantitative finding that viewing the ultrasound image mediated the association between the law and continuing a pregnancy, we focus our analysis below on how respondents described the effects of ultrasound *viewing*, with attention to how that experience fit into the broader context of their lives and decision-making.

#### Effects of ultrasound viewing

All four of the respondents who declined to view their ultrasound image proceeded to abortion and, for the most part, those who did view their ultrasound image did not talk about viewing as having an impact on their decision to have an abortion. For example, one woman who viewed her ultrasound image because she thought it was “interesting” to see, reported that viewing had no impact on her decision to have an abortion: “Didn't change my mind at all or my feelings or anything like that.” Another, who explained she chose to view in order to inform her decision-making process, was similarly unaffected by viewing, stating that it had, overall, no effect: “I could probably have gone away without seeing or not seeing it. I don’t think it affected anything.” Instead, respondents identified other factors as having a larger impact on their decision to have an abortion, such as the difficulty of securing funds to cover the cost of the abortion. Respondents who went to great lengths to overcome these factors—like, for example, the respondent who spent two days calling an abortion fund, seeking financial aid, before getting through and receiving support—were particularly unaffected by viewing. Across the respondents, as these examples demonstrate, the most common reported reaction to viewing was a neutral one; for most women, viewing had no emotional effect or impact on their decision to have an abortion.

Eight respondents, however, did report that viewing had an effect on them. Perhaps unexpectedly, for five of these eight, viewing solidified their decision to have an abortion. One explained:

I said, “Yeah, I want to see it.” And then, I looked at it, and it's just a sac, that's it. And here it is right here. And then, yeah, that was it. That's how I knew I was ready [to have the abortion.] And I realized when I saw it, I wasn't emotionally connected to that as a child yet, so I was able to know from that point when I looked at the ultrasound I didn't feel bad. I was like, that's—you know—people—I was okay with it. So, I do think that was a positive experience that I was well enough to look at it and say, "Yeah, I want to see it, and yeah, I'm not having it.”

Later in the interview, she summed up the effect of viewing, saying, “I just feel like looking helped me kind of accept, you know, be certain [about choosing abortion].”

For another woman, who reported choosing to view in order to confirm her decision to have an abortion, viewing did stir up some negative emotions: it reminded her of viewing the ultrasounds for her two existing children. Nonetheless, she said that experiencing those feelings did not make her waver on her decision to have an abortion. She explained, “Regardless of what I've seen, how I feel, even if I was to feel like I should keep it, at the end of the day, my mind's set that I shouldn't keep it, because I'm not ready.”

Two of these respondents who described viewing their pre-abortion ultrasound image as having an emotional effect on them, however, said viewing contributed to their decision to continue the pregnancy. One respondent, for example, who was 10 weeks pregnant, showed up for her abortion appointment (which, per Wisconsin law, is at least 24 hours after her first appointment at the clinic) but left without obtaining the procedure, having decided to continue the pregnancy. She attributed some of her decision to continue the pregnancy to viewing her pre-abortion ultrasound scan at the abortion facility as well as viewing ultrasound images she received at a nearby hospital prior to her appointment at the abortion facility. She said, “maybe looking at all the ultrasounds made me change my mind.” Deeper consideration of her experience, however, points to ongoing ambivalence about choosing abortion. She explained that, at the time of her first appointment (i.e. the ultrasound appointment), she was “not at all” sure abortion was the right decision for her. She elaborated:

Basically just listening to other people was—it kind of brought me there to even consider the abortion. It was never a thought of my own. So, and I finally listened to myself I felt like it [abortion] wasn’t for me, and I knew that I wasn’t going to be happy with the decision.

She continued, explaining that abortion was her boyfriend’s preference:

He didn’t want another child. Didn’t want anything to do with raising another child. So, it [abortion] was just basically his idea—a lot of it. Kind of felt like I didn’t really have a choice, and it was more of a thing that I would have been forced to do.

At the same time, her mother disapproved of her plan to have an abortion and offered to adopt the baby after it was born, allowing the respondent to have a role in the baby’s life. She explained that viewing the ultrasounds made her ask herself, “why do I have to abort my child when I know I have the option to keep it?” These thoughts, however, were not brought on by viewing the ultrasound image; she said, “I was kind of already having those thoughts […] it [abortion] was just something that I was never comfortable with.” She had not, however, articulated this hesitation to a member of the clinic staff. When they asked her if she wanted an abortion, she said she did and that it was her decision.

Another respondent, already a mother of five children between 8-years-old and 5-months-old, also attributed her decision to continue this pregnancy to viewing the ultrasound image. She described viewing her ultrasound image as a turning point in her pregnancy decision-making. This respondent had not considered abortion with any of her previous pregnancies, even for a second, but this time was different because her youngest was so young and her most recent pregnancy had been difficult, with several medical complications. She worried about her ability to have a safe pregnancy. Still, she was unsure about abortion: “It was probably a back and forth for a long time, which was probably why there was such a huge gap [of seven weeks] about when I found out about being pregnant and actually when I went to the clinic. It was a lot of back and forth.” When asked how certain she was at the time of her first appointment that abortion was the right decision for her, she replied, “I don’t think I was at all.” She did not, however, share this uncertainty with any clinic staff members, instead conveying to them that she was firm in her decision to have an abortion.

Looking at the ultrasound image of her 16-week pregnancy clarified her decision and resolved her uncertainty:

It made me feel like I finally knew a little bit of how I felt about the whole situation. Before, I was unsure if it's the right thing to do. And, then when I—like, she was kicking her feet and the hand was going across the face, and all I could think was like, how do you do something like this? It's clearly alive, it's clearly moving around. Like, how? It's mine. That's my baby.

When she spoke to the father of her children soon after, he agreed and they jointly decided to continue the pregnancy and raise this child.

Finally, one respondent reported that she experienced happiness at viewing the ultrasound image: “looking at the screen didn’t hurt me, actually. It made me happy, at least for a brief moment.” Unlike any of the other women interviewed, this respondent was sure that she did *not* want to have an abortion. In fact, she wanted desperately to keep the pregnancy, but found herself in a complex legal situation wherein having the baby would likely result in her boyfriend going to jail. At the time of the interview, she had not scheduled her abortion appointment, saying, “I’m still holding out hope [that I can continue this pregnancy].” By her account, viewing did not affect her abortion decision. It did not add to or even impact her desire to continue the pregnancy. Like the women who were certain that abortion was the right decision for them, this respondent experienced viewing as unrelated to her abortion decision-making. Indeed, looking offered her a memory she could think back on in the future if she proceeds with the abortion. She said, “[by looking] I, you know, can still have that little part of me because for a few months’ time, you know, that was my baby.”

## Discussion

This is the first study to explore the effects of a mandated pre-abortion ultrasound viewing law on women, including whether it affects their decision to proceed with the abortion, using a mixed methods study design. In this study, chart data showed that Wisconsin’s mandatory pre-abortion ultrasound viewing law was associated with a statistically significant and robust, but small, increased likelihood of continuing pregnancy, regardless of a woman’s certainty about her abortion decision. However the in-depth interview findings demonstrate that the effect of viewing on a woman’s decision to continue her pregnancy is best understood with attention to the broader context of her life circumstances. The multivariable analysis of the chart data was consistent with the qualitative finding that other factors were more important to her abortion decision. The innovative methods used in this paper integrating qualitative and quantitative research methods and analyses allow us a broad look at the question of how a mandatory ultrasound viewing law impacts women’s abortion decisions.

The quantitative findings confirm the part of the conceptual model where pre-abortion ultrasound viewing mediates the relationship between the law and women’s decisions to proceed to abortion or continue their pregnancies. In fact, viewing fully mediates the association so that the law has no independent impact on women’s abortion decisions. We expected to also find that decision certainty moderates the impact of pre-ultrasound viewing on continuing pregnancy. While we were unable to formally test this association due to small numbers of uncertain women who did not view the image, stratified tabulations do not support this moderation effect. They suggest that the effect of mandated viewing impacts all women regardless of their decision certainty. This finding differs from a previous study in a voluntary context [15], where ultrasound viewing had a small impact only among women who were not certain that abortion was the right decision for them. Nevertheless, the current study showed that low decision certainty has strong associations with continuing pregnancy, and this is consistent with the previous study done in the context of voluntary ultrasound viewing [15].

The in-depth interviews offer some insight into this apparent divergence from the conceptual model and prior studies. In the interview data, it was only the two women who were uncertain about choosing abortion who described viewing the ultrasound image as having an impact on them, consistent with previous research that low decision certainty plus viewing is associated with continuing the pregnancy. Their complex personal stories suggest, moreover, that they may have been seeking to justify choosing to continue the pregnancy. Citing ultrasound viewing as causing an attachment to the pregnancy, despite in at least one respondent’s case having previously viewed a high quality image at a hospital, may have enabled these women to persuade themselves and those in their lives that continuing the pregnancy was the right decision for them. Neither of these women, however, communicated their uncertainty about abortion to clinic staff, suggesting that the facility’s question on decision certainty may not have completely captured the variability and nuance in decision certainty among this population. Thus the quantitative measure of decision certainty was perhaps imprecise. As a dichotomous measure, it did not capture fine distinctions among levels of decision certainty. As one indicator, the proportion of women who were uncertain in this study appears lower than in some previous studies of women seeking abortion [15, 23]. This would explain why we found a significant effect of the law even among those who reported being firm in their decision.

Other factors identified in the quantitative analysis that were associated with proceeding to abortion bear discussion. That the proportion of women who qualified for assistance from an abortion fund dropped between pre-law and post-law periods, together with the finding that women who qualified for assistance from an abortion fund were more likely to proceed to abortion than those who would have to pay fully out of pocket, suggests that the drop in abortion funds available may have contributed to the increase in the percentage of women continuing pregnancy between pre- and post-law periods. Indeed reports from the clinic director confirm that due to funding constraints among abortion funds, fewer women were granted abortion funding in the post-law period. Abortion funds provide women with needed assistance to surmount financial barriers and make it possible to have a wanted abortion. Research has found that cost is a major barrier to obtaining a wanted abortion for women [31–33]. Future research should examine this relationship further.

Women reporting that their partners were the dominant decision-makers in their relationships were more likely to continue their pregnancies which demonstrates that men can have a strong influence on women’s abortion decisions. While the literature finds that most men are supportive of women’s abortion decisions [34, 35], it also finds that the small proportion of men who are controlling or violent are less likely to support women’s reproductive decisions [34, 36, 37].

There are several strengths of our study. The qualitative sample includes a diverse group of women and the quantitative portion avails of a sufficiently large sample size. Our measure of viewing assessed viewing the printout as well as the image on screen to capture the consumption of any ultrasound image; no other study has examined the effects of the printout of the image. Further research is needed to understand whether and how women experience the printout differently from the image on screen.

There are a few limitations, however. First, in addition to viewing, the law also required providers to describe fetal development to all patients in the post-law period which may also have contributed to the effects seen among women in decisions to continue their pregnancies, but this study is unable to tease apart such effects. Second, women who were eligible for an abortion but did not return for an abortion were assumed to have continued the pregnancy, although it is possible that some obtained an abortion at another facility. There is no reason to believe that there was an increase in the percentage of women who went elsewhere for an abortion after the law, as there were no new abortion services over the study period. Third, the interview data were collected exclusively in the post-law period, so we cannot know whether their reports of any effects (or lack thereof) of viewing are specific to the mandatory viewing setting. Finally, because the chart abstraction and qualitative interviews took place concurrently, the interviews did not probe unexpected areas the quantitative analysis identified as of interest, such as the role of funding and whether women self-identified as the primary decision-maker. Future research should examine these points further.

Together, these qualitative and quantitative analyses find that Wisconsin’s mandatory ultrasound viewing law caused an increase in viewing rates and, in turn, slightly increased the rate of continuing pregnancies. However, for the vast majority of women, the law does not change their minds about abortion; other factors have important roles in their decision-making.

Even with these robust data, we do not fully understand the precise mechanism behind “viewing” that is having the effect. Given that the image itself is different depending on the stage of fetal development, the image quality of the machine at this facility was not high, and all women—even those who averted their eyes—heard a verbal description of the image, we are unable to tease out *how* the law mandating viewing impacted decision-making for some, but not all, women. Indeed, in the in-depth interviews, the women who articulated an effect of viewing also described other factors that were personally significant to their decision-making, making it impossible to isolate anything specific about viewing the image. One possible mechanism we posit is that the effect of viewing is a culmination of existing social pressure to continue pregnancies, the existence of which is evidenced by the fact that a mandatory ultrasound viewing law has legislative support. It is important to consider the way a broader social environment that condones mandatory viewing may itself condition women’s abortion decisions. With data from only a single state, our results may not be generalizable to other states with the same law, but we do expect that these findings would be consistent in other states with laws that are hostile to abortion rights. In such an environment the process of having the ultrasound image described and displayed may be the tipping point that leads a woman who was in the process of making her decision about whether to have an abortion decide to continue her pregnancy. The question of whether this cumulative social pressure to continue a pregnancy is coercive is important as evidence shows negative consequences for women who do not receive wanted abortions [38, 39].

In contexts where viewing the ultrasound image is decided by the patient, a significant minority of women seeking abortions already choose to view their ultrasound image. At the same time, in the absence of such a law the majority of women choose not to view for a variety of personal reasons [17]. Ultrasound viewing is not integral to the medical provision of abortion care. Thus, laws about whether to force women to view their ultrasounds are not a question of quality of care but instead are a question of values regarding whether the state should use legislation to attempt to influence women’s abortion decisions. Together, these findings suggest that we should think about viewing not as a standalone experience, but as mediated by the broader social environment and the context of women’s lives and circumstances.

## Supporting information

S1 Data Deidentified individual-level chart data. https://doi.org/10.7272/Q65H7D63.
(XLS)

S2 Data Monthly aggregate data. https://doi.org/10.7272/Q65H7D63.
(XLS)

S1 Text Supplemental information on methods. (DOCX)
