# Supplementary material for: Evaluating the impact of a mandatory pre-abortion ultrasound viewing law: A mixed methods study
Source: PLoS One. 2017 Jul 26;12(7):e0178871. doi: 10.1371/journal.pone.0178871 (PMC5528259; doi:10.1371/journal.pone.0178871)
Supplement: S1 Text — (DOCX) [file pone.0178871.s003.docx]

**Supplemental Information on Methods**

**Methods for Medical Chart Data**

The UCSF research team provided a full day of on-site training to each of the three data abstractors in the standardized data abstraction protocol, which covered data abstraction methods, basic research principles, ethical conduct of research, and detailed instructions for all data abstraction fields. Abstractors were given a training manual that they kept on-hand as they abstracted data. All data were abstracted from paper charts and entered into and immediately saved on an encrypted and HIPAA-compliant electronic platform that was only accessible to the research team. Each abstractor received an approximately equal balance of pre- and post-law charts and was instructed and reminded to enter all data and clinical notes as they appeared in the chart and to use notes fields to explain any errors or discrepancies noticed. Outside of the notes fields, abstractors were instructed not to interpret the data, even if they thought there was an error. Abstractors checked for data entry errors by performing regular checks on charts chosen by the research team at random. Errors were corrected as they were found and prevented by more frequent checks and additional training and clarification. Abstraction occurred between March and September 2015.

**Methods for In-depth interviews**

For one week during the recruitment period, a member of the study team was on-site. For that week, interested participants were screened in-person and, if eligible, verbally consented in-person by the research assistant.

Respondents were also asked about their experience at the facility with wait times, counseling, and interaction with the staff; pregnancy background, including the circumstances of conception, discovery of pregnancy, and interactions, if any, with others about the pregnancy. Additionally, we collected sociodemographic information including age, race, weeks gestation at their first appointment, and number of children. Interviews ranged from just under 40 minutes to one hour and 30 minutes in length, averaging about 60 minutes.
